# Kinematic alignment without femoral cartilage‐wear compensation for apex‐distal joint line obliquity: Effects on component alignment

**DOI:** 10.1002/jeo2.70557

**Published:** 2025-11-14

**Authors:** Tsutomu Maeda, Theodore Derek Vernon Cooke, Mitsuhiko Kubo, Kazutaka So, Shinji Imai

**Affiliations:** ^1^ Department of Orthopaedic Surgery Shiga General Hospital Moriyama Shiga Japan; ^2^ Department of Orthopaedic Surgery Shiga University of Medical Science Otsu Shiga Japan; ^3^ School of Rehabilitation Sciences Ǫueen's University Kingston Ontario Canada; ^4^ Department of Sports and Musculoskeletal Science Shiga University of Medical Science Otsu Shiga Japan

**Keywords:** cartilage‐wear compensation, CPAK classification, kinematic alignment, joint line obliquity, total knee arthroplasty

## Abstract

**Purpose:**

Pronounced apex‐distal joint line obliquity (JLO) complicates total knee arthroplasty (TKA) by challenging patellofemoral tracking and medial tibial bone support. Joint line obliquity–modified kinematic alignment (JLO‐KA)—a selective modification of kinematic alignment (KA) that omits femoral cartilage‐wear compensation and reallocates correction to the tibial side—was developed. This study quantified postoperative component and limb alignment with JLO‐KA versus true KA.

**Methods:**

Retrospective comparison of 20 JLO‐KA knees and 15 true‐KA knees with preoperative apex‐distal JLO (CPAK I–III). Pre‐/postoperative computed tomography (CT) measured lateral distal femoral angle (LDFA), medial proximal tibial angle (MPTA), femoral component rotation (FCR), arithmetic hip–knee–ankle angle (aHKA), and JLO; postoperative Coronal Plane Alignment of the Knee (CPAK) distribution was analysed (*Δ* = JLO‐KA minus true KA).

**Results:**

Groups were similar at baseline: preoperative LDFA 87.7° versus 87.6°, MPTA 83.5° versus 83.5°, aHKA −4.3° versus −4.1°, JLO 171.2° versus 171.2° (all *p* > 0.05). Postoperatively, JLO‐KA increased LDFA to 90.4° ± 2.3° versus 87.0° ± 1.9° (*Δ* = +3.4°, 95% confidence interval [CI]: 1.9–4.8; *p* < 0.0001), MPTA to 88.0° ± 1.4° versus 85.6° ± 2.0° (*Δ *= +2.4°, 1.1–3.7; *p* = 0.0015), and FCR to 3.1° ± 2.0° versus 0.1° ± 2.0° (*Δ *= +2.9°, 1.5–4.3; *p* = 0.0002), while aHKA was similar (−2.4° ± 3.1° vs. −1.4° ± 2.8°; *Δ* = −1.0°; *p* = 0.324). JLO was closer to neutral with JLO‐KA (178.4° ± 2.2° vs. 172.7° ± 2.8°; *Δ* = +5.8°; *p* < 0.001). Neutral‐JLO CPAK types (IV–VI) occurred in 16/20 (80%) versus 2/15 (13%) (*p* = 0.00013). The restricted KA 90° ± 5° range for LDFA and MPTA was met by 19/20 (95%) versus 7/15 (47%) (*p* = 0.0019).

**Conclusion:**

Reallocating cartilage‐wear compensation from the medial femur to the medial tibia within the same calliper‐verified workflow reduced femoral valgus, limited tibial varus, and increased femoral external rotation by ≈3° while maintaining aHKA. Shifts were consistent with lateralizing the prosthetic trochlear groove and preserving medial tibial bone support, positioning JLO‐KA as a targeted option for apex‐distal knees (CPAK I–III).

**Level of Evidence:**

Level III, retrospective comparative study.

**Abbreviations**
3D: three‐dimensional
aHKA: arithmetic hip–knee–ankle angle
CI: confidence interval(s)
CPAK: Coronal Plane Alignment of the Knee
CR: cruciate retaining
CT: computed tomography
EZR: Easy R (Statistical Software Interface)
FCR: femoral component external rotation
FJS: Forgotten Joint Score
HKA: hip–knee–ankle angle
ICC: intraclass correlation coefficient
JLO: joint line obliquity
JLO‐KA: joint line obliquity–modified kinematic alignment
KA: kinematic alignment
LDFA: lateral distal femoral angle
MA: mechanical alignment
MPTA: medial proximal tibial angle
MS: medial stabilized
PROMs: patient‐reported outcome measures
PS: posterior stabilized
rKA: restricted kinematic alignment
SD: standard deviation
TKA: total knee arthroplasty

## INTRODUCTION

Joint line obliquity (JLO) is the coronal inclination of the tibiofemoral articular surface relative to the limb's mechanical axis, quantified as the sum of the lateral distal femoral angle (LDFA) and medial proximal tibial angle (MPTA); by definition, 180° denotes a neutral joint line, and lower values indicate an apex‐distal pattern [19]. An apex‐distal JLO is commonly observed in alignment surveys [19] and is more prevalent in Asian populations [15, 25, 32]. In total knee arthroplasty (TKA), calliper‐verified kinematic alignment (‘true KA’) matches the implant to the resected bone with cartilage‐wear compensation to reproduce the prearthritic joint surfaces [7, 24]. Accordingly, true KA typically restores the patient‐specific obliquity [7, 24], which is often apex‐distal [15, 19, 25, 32].

However, in knees with pronounced apex‐distal JLO, reproducing the joint line with true KA can present two practical concerns:

(1) Patellofemoral tracking: True KA does not fully restore the native trochlear anatomy [27], and comparative studies have shown altered patellofemoral alignment relative to mechanical alignment (MA) [22]. Because femoral component positioning strongly influences patellar tracking [17, 26], medialization of the prosthetic trochlear groove—especially in CPAK (Coronal Plane Alignment of the Knee) types III and VI and to a lesser extent in Types I and II—has been associated with lower Forgotten Joint Scores (FJS) [12].

(2) Medial tibial bone support: When medial resection extends beyond the subchondral plate, the residual bone may be insufficient to support the tibial baseplate [11, 23]. Greater varus alignment is associated with higher medial tibial stresses [23], and varus tibial component alignment has been associated with greater tibial baseplate migration [31].

Joint line obliquity–modified kinematic alignment (JLO‐KA) is proposed as a selective modification of true KA to address these concerns. In line with the concept that ‘every knee has its own envelope of alignment’ [6], alignment strategies may be individualized within safe boundaries [1, 2, 3, 4, 16]. Within a calliper‐verified workflow [7, 24], the standard 2‐mm cartilage‐wear compensation used in true KA was omitted on the medial femoral side and reallocated to the medial tibial plateau. This modification is intended to lateralize the prosthetic trochlear groove [12, 17] and preserve medial tibial bone support, consistent with biomechanical stress and migration data [23, 31].

The effects of reallocating the 2‐mm cartilage‐wear compensation on alignment metrics and CPAK transitions are unknown. It was hypothesized that, compared with true KA, JLO‐KA would produce approximately 3° shifts—greater femoral component external rotation (FCR) and less femoral valgus and tibial varus—while maintaining the arithmetic hip–knee–ankle angle (aHKA; MPTA − LDFA, positive indicates valgus). Accordingly, the objective of this study was to characterize the alignment consequences of omitting femoral cartilage‐wear compensation within a calliper‐verified workflow: namely, shifts in component‐alignment angles (LDFA, MPTA and FCR), overall alignment metrics (JLO and aHKA) and the distribution of CPAK types [19].

## PATIENTS AND METHODS

### Study design

A single‐centre, single‐surgeon, retrospective comparative cohort study was conducted from June to December 2023.

### Patient selection

Consecutive primary TKAs were identified from the institutional electronic medical record. Eligibility required preoperative CPAK Types I–III (apex‐distal), medial‐compartment osteoarthritis and availability of both pre‐ and postoperative whole‐limb computed tomography (CT). Of 51 knees screened, 15 were excluded—10 for missing postoperative CT, 4 with lateral‐compartment osteoarthritis and 1 with post‐traumatic osteoarthritis. One knee initially scheduled for JLO‐KA was preoperatively identified as CPAK Type V and treated with true KA; this case was excluded from analysis. The final cohort comprised 35 knees: 20 treated with JLO‐KA and 15 with true KA. Postoperative CPAK was analysed as an outcome and was not used for eligibility.

### Technique allocation and implant selection

Technique allocation followed an implant‐dependent protocol. JOURNEY II CR (Smith & Nephew) cases used true KA because the femoral component incorporates approximately 3° of distal lateral inclination that addresses JLO [21]. ATTUNE CR/MS/PS (DePuy Synthes) adopted JLO‐KA from the outset to moderate femoral valgus and limit tibial varus. GMK Sphere (Medacta International) transitioned from true KA to JLO‐KA mid‐period, appearing in both cohorts. All procedures were performed by a single experienced surgeon. The unequal group sizes reflect consecutive enrolment during sequential protocol implementation.

### Surgical technique

**Figure 1:**
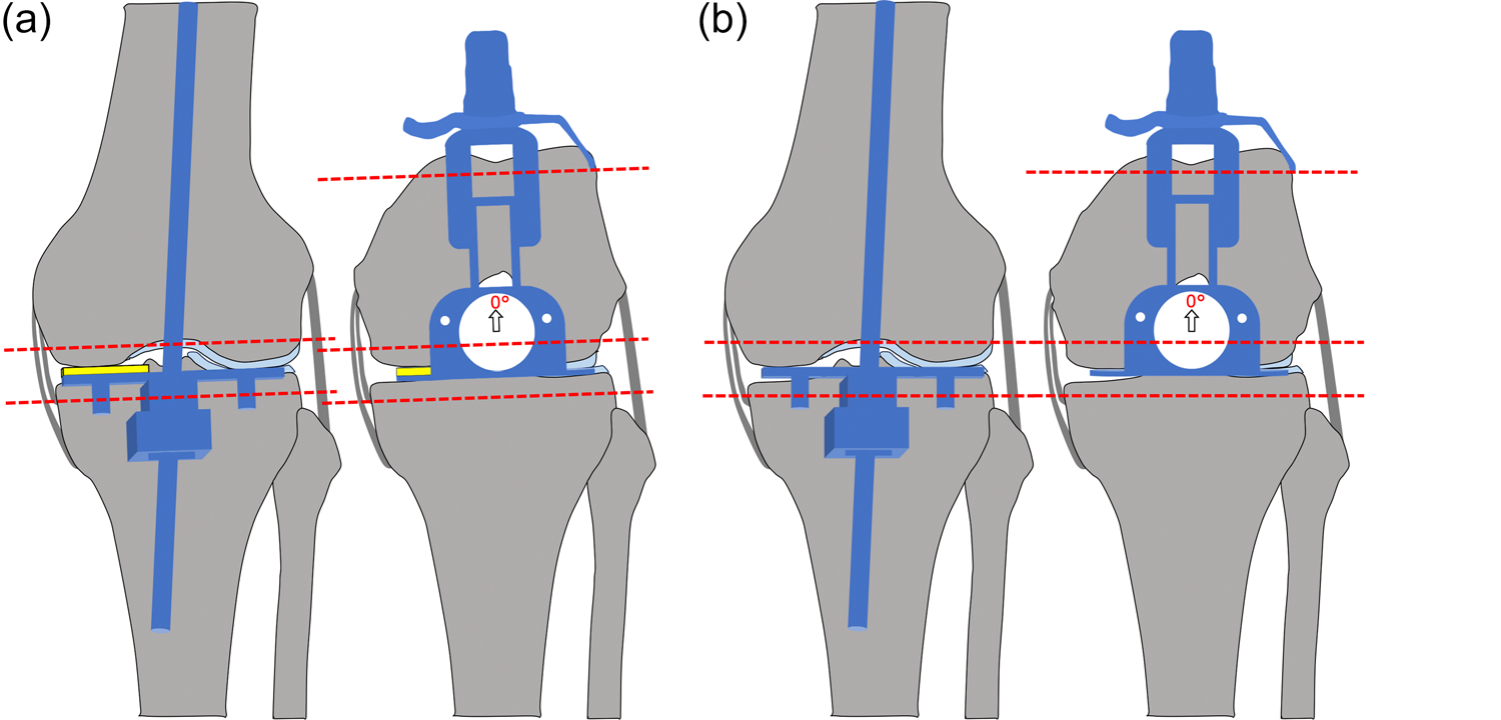
Comparison of femoral resections between true KA (a) and JLO‑KA (b): schematic of distal and posterior femoral cuts using conventional instruments. Grey = bone; light blue = cartilage; yellow = 2‐mm shim. True KA (a): a 2‐mm medial shim compensates for cartilage wear to reproduce the prearthritic joint surface with symmetric femoral bone resection thickness, calliper‐verified to the implant thickness [7, 24]. JLO‑KA (b): shim omitted; medial femoral bone resection is 2 mm greater than in true KA, yielding less valgus and greater external rotation of the femoral component than with true KA. JLO‐KA, joint line obliquity–modified kinematic alignment; KA, kinematic alignment.

**Figure 2:**
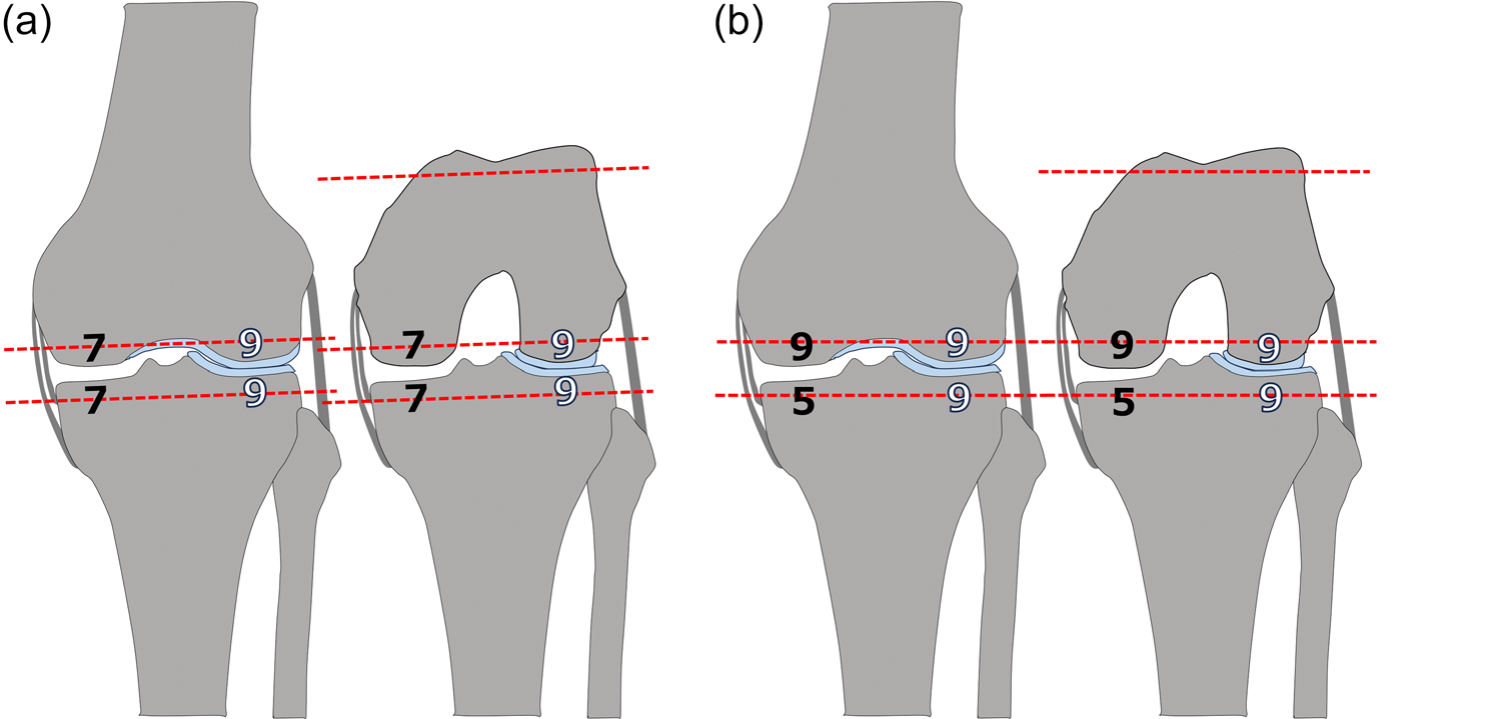
Comparison of bone‐resection thickness between true KA (a) and JLO‑KA (b) at the distal/posterior femur and proximal tibia. Grey = bone; light blue = cartilage. Black numbers (mm) denote bone‐only resection thickness; white numbers (mm) denote resection thickness including cartilage. True KA (a): a 2‐mm medial femoral shim compensates for cartilage loss; 7 mm of bone is resected from each femoral condyle, with the lateral cartilage included to match a 9‐mm implant [7, 24]. JLO‑KA (b): shim omitted; 9 mm is resected equally from both femoral condyles—bone only medially, cartilage plus bone laterally—and the medial tibial resection is reduced by 2 mm, shifting compensation for medial femoral cartilage wear to the tibia. JLO‐KA, joint line obliquity–modified kinematic alignment; KA, kinematic alignment.

For the schematics (Figures 1, 2 and 2), a generic 9‐mm component thickness was assumed for the distal/posterior femur and proximal tibia in both techniques.

### True KA

Medial femoral cartilage‐wear compensation (2 mm) was applied so that resection thickness equalled component thickness, reproducing the prearthritic joint surfaces [7, 24] (Figures 1, 2 and 2a). True KA with JOURNEY II was calliper‐verified and accounted for the implant's asymmetric condylar and insert thicknesses when determining resection amounts [10].

### JLO‐KA

Medial femoral cartilage‐wear compensation was omitted and reallocated to the medial tibial plateau. In a typical pattern with full‐thickness medial cartilage loss and intact lateral compartments:

#### Femur

Distal and posterior resections were 9 mm each—bone‐only medially and cartilage‐plus‐bone laterally. Residual medial posterior cartilage was removed, and the medial posterior condyle was referenced to bone; no additional external rotation was dialled into the guide (0°). This yielded approximately 2°–3° less femoral valgus and approximately 2°–3° more femoral external rotation than true KA (Figures 1b and 2b).

#### Tibia

A 5‐mm medial resection (subchondral bone only) and a 9‐mm lateral resection (cartilage and bone) were performed, corresponding to a 2‐mm medial under‐resection relative to true KA, resulting in approximately 2°–3° less tibial varus (Figure 2b).

### Imaging and measurements (alignment analysis)

**Figure 3:**
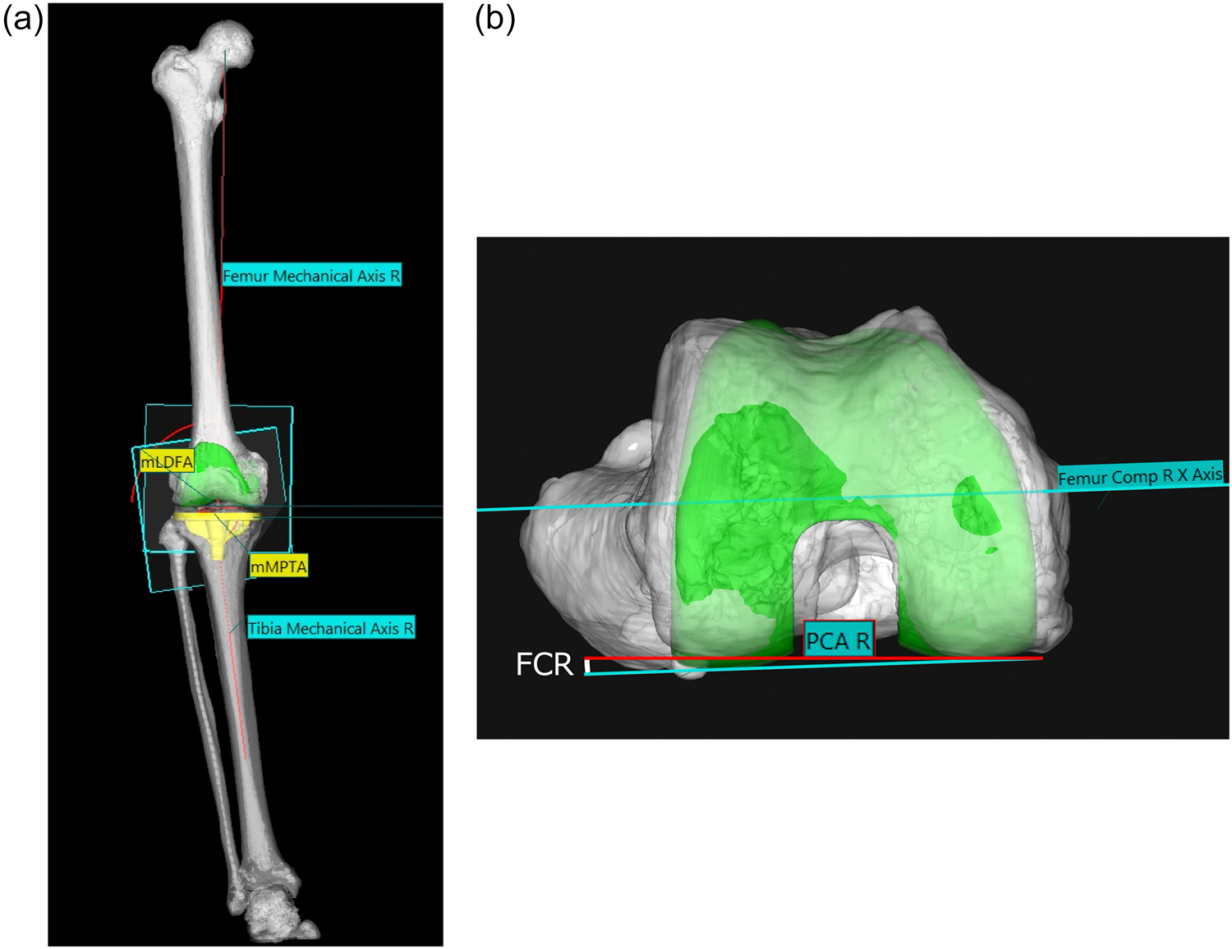
CT‐based measurement workflow and angle definitions. Pre‐ and postoperative whole‐limb CTs were matched in ZedKnee via automated 2D/3D image matching. LDFA: angle between femoral mechanical axis and distal joint line tangent of the femoral component. MPTA: angle between tibial mechanical axis and proximal joint line tangent of the tibial component. JLO = LDFA + MPTA; aHKA = MPTA − LDFA. FCR: femoral component rotation relative to the posterior condylar line (external rotation positive). Measurements follow MacDessi et al. for coronal metrics and ZedKnee's CT‐based workflow described in prior validation studies [19, 30, 33]. 2D/3D, two‐/three‐dimensional; aHKA, arithmetic hip–knee–ankle angle; CT, computed tomography; FCR, femoral component external rotation; JLO, joint line obliquity; LDFA, lateral distal femoral angle; MPTA, medial proximal tibial angle.

Pre‐ and postoperative coronal and rotational alignment were assessed with whole‐limb CT and 3D modelling software (ZedKnee, LEXI Inc.) [30, 33] (Figure 3). Parameters included LDFA, MPTA, FCR (external rotation positive relative to the posterior condylar line), aHKA ( = MPTA − LDFA; positive indicates valgus), and JLO ( = LDFA + MPTA) [19]. Values were plotted on the CPAK grid and classified according to CPAK thresholds: JLO—neutral 177°–183°, apex‐distal <177°, apex‐proximal >183°; aHKA—neutral −2° to +2° (varus < −2°, valgus > +2°) [19]. For JOURNEY II, 3° was subtracted from the distal femoral bone–implant interface to account for the component's built‐in lateral inclination when reporting CT angles [21].

### Measurement process and reliability

All measurements were performed by a single assessor (orthopaedic surgeon, >20 years' experience) using the software's automated two‐/three‐dimensional (2D/3D) image‐matching workflow. Angles were computed within a consistent CT‐based coordinate system. Formal interobserver or intraobserver reliability testing was not performed in this cohort; this workflow has demonstrated reproducibility with intraclass correlation coefficient (ICC) values of 0.99–1.00 in prior validation studies [30, 33].

### Outcomes of interest

The primary outcome was postoperative LDFA. Secondary outcomes were postoperative MPTA, FCR, JLO, aHKA and CPAK type distribution.

### Statistical analysis

Normality was assessed with the Shapiro–Wilk test (all *p* > 0.05) and *Q*–*Q* plots; therefore, parametric tests were used. Continuous variables were compared with Welch's *t*‐test (*n* = 20 vs. 15). Categorical variables (sex, laterality) were compared with Fisher's exact test. Postoperative LDFA (primary outcome) was tested at *α* = 0.05 without multiplicity adjustment. For secondary outcomes (MPTA, FCR, JLO and aHKA), the Holm–Bonferroni correction was applied; adjusted *p* values are reported. Effect sizes are presented as mean differences (JLO‐KA minus true KA) with 95% confidence intervals (CIs). No imputation was performed (complete‐case analysis).

Categorical endpoints (e.g., proportion in the neutral‐JLO row [CPAK IV–VI] and proportion within the restricted kinematic alignment (rKA) boundary [LDFA and MPTA 85°–95°]) were compared with two‐sided Fisher's exact tests. Proportion 95% CIs were calculated with the Wilson method, and 95% CIs for differences in proportions with the Newcombe method. These analyses were exploratory; *p* values were not multiplicity‐adjusted.

No a priori power analysis was conducted. To contextualize precision, minimum detectable differences (80% power, two‐sided *α* = 0.05) were estimated from the observed pooled standard deviations (SDs) and group sizes (*n* = 20 vs. 15): LDFA ≈ 2.0°, MPTA ≈ 1.6°, FCR ≈ 1.9°, JLO ≈ 2.4°, and aHKA ≈ 2.8°. All analyses were conducted using EZR (Easy R) [13].

## RESULTS

### Baseline characteristics

Thirty‐five TKAs were analysed (JLO‐KA, *n* = 20; true KA, *n* = 15). Age and body mass index (BMI) were comparable between groups (Table 1). Sex and laterality were also similar (female: 15/20 [75.0%] vs. 9/15 [60.0%], *p* = 0.47; right knees: 8/20 [40.0%] vs. 9/15 [60.0%], *p* = 0.3150). By design, implant use differed: the JLO‐KA group used ATTUNE (*n* = 19) or GMK Sphere (*n* = 1), whereas the true‐KA group used JOURNEY II (*n* = 10) or GMK Sphere (*n* = 5) (see Methods section).

**Table 1:** Baseline demographics and CT‐based alignment parameters before and after TKA: JLO‐KA versus true KA.

Variable	JLO‐KA (*n* = 20)	True KA (*n* = 15)	Difference (JLO‐KA − true KA)	*p* value
Demographics				
Age, years	76.2 (6.9) [72.9 to 79.4]	73.7 (4.9) [71.0 to 76.4]	+2.5 [−1.6 to 6.5]	0.2242
Female, *n*/*N* (%)	15/20 (75%)	9/15 (60%)	+15 pp [−27 to 53]	0.4674
BMI, kg/m^2^	24.6 (4.0) [22.7 to 26.5]	26.9 (4.0) [24.6 to 29.1]	−2.3 [−5.1 to 0.5]	0.1083
Right side, *n*/*N* (%)	8/20 (40%)	9/15 (60%)	−20 pp [−58 to 26]	0.3150
Preoperative alignment (CT)				
LDFA, °	87.7 (1.9) [86.8 to 88.6]	87.6 (2.1) [86.5 to 88.8]	+0.1 [−1.3 to 1.5]	0.8891
MPTA, °	83.5 (2.1) [82.5 to 84.4]	83.5 (2.0) [82.5 to 84.6]	−0.1 [−1.5 to 1.3]	0.8936
aHKA, °	−4.3 (3.2) [−5.8 to −2.8]	−4.1 (3.5) [−6.0 to −2.1]	−0.2 [−2.5 to 2.2]	0.8696
JLO, °	171.2 (2.5) [170.0 to 172.3]	171.2 (2.1) [170.0 to 172.3]	+0.0 [−1.6 to 1.6]	0.9957
Postoperative alignment (CT)				
LDFA, °	90.4 (2.3) [89.3 to 91.5]	87.0 (1.9) [86.0 to 88.1]	+3.4 [1.9 to 4.8]	<0.0001
MPTA, °	88.0 (1.4) [87.3 to 88.7]	85.6 (2.0) [84.5 to 86.8]	+2.4 [1.1 to 3.7]	0.0015
aHKA, °	−2.4 (3.1) [−3.8 to −0.9]	−1.4 (2.8) [−2.9 to 0.1]	−1.0 [−3.0 to 1.0]	0.3243
JLO, °	178.4 (2.2) [177.4 to 179.5]	172.7 (2.8) [171.1 to 174.2]	+5.8 [3.9 to 7.6]	<0.0001
FCR, °	3.1 (2.0) [2.1 to 4.0]	0.1 (2.0) [−1.0 to 1.3]	+2.9 [1.5 to 4.3]	0.0002

*Note*: Values are mean (SD) [95% CI] unless stated. Difference = JLO‐KA − true KA. Continuous outcomes were compared with Welch's *t*‐test. *p* Values are Holm‐adjusted for secondary postoperative outcomes (MPTA, aHKA, JLO, FCR). Categorical variables were compared with Fisher's exact test; differences are absolute percentage‐point (pp) differences with Newcombe 95% CI. Angles in degrees; aHKA is positive for valgus, and FCR is positive for external rotation.

Abbreviations: aHKA, arithmetic hip–knee–ankle angle; BMI, body mass index; CI, confidence interval; CT, computed tomography; FCR, femoral component external rotation; JLO, joint line obliquity; KA, kinematic alignment; LDFA, lateral distal femoral angle; MPTA, medial proximal tibial angle; SD, standard deviation; TKA, total knee arthroplasty.

### Alignment analysis

Preoperative alignment parameters did not differ between groups (all *p* > 0.05; Table 1). Postoperatively, the JLO‐KA group showed higher LDFA, MPTA and FCR than the true KA group (ΔLDFA = +3.4° [95% CI: 1.9–4.8], *p* < 0.0001; ΔMPTA = +2.4° [1.1–3.7], *p* = 0.0015, Holm‐adjusted; ΔFCR = +2.9° [1.5–4.3], *p* = 0.0002, Holm‐adjusted; *Δ* denotes JLO‐KA minus true KA). The aHKA was similar between groups (−2.4° ± 3.1° vs. −1.4° ± 2.8°; *Δ* = −1.0° [−3.0 to 1.0], *p* = 0.324, Holm‐adjusted). The JLO angle was closer to 180° (neutral) with JLO‐KA (178.4° ± 2.2°) than with true KA (172.7° ± 2.8°; *Δ* = +5.8° [3.9–7.6], *p* < 0.001, Holm‐adjusted).

**Figure 4:**
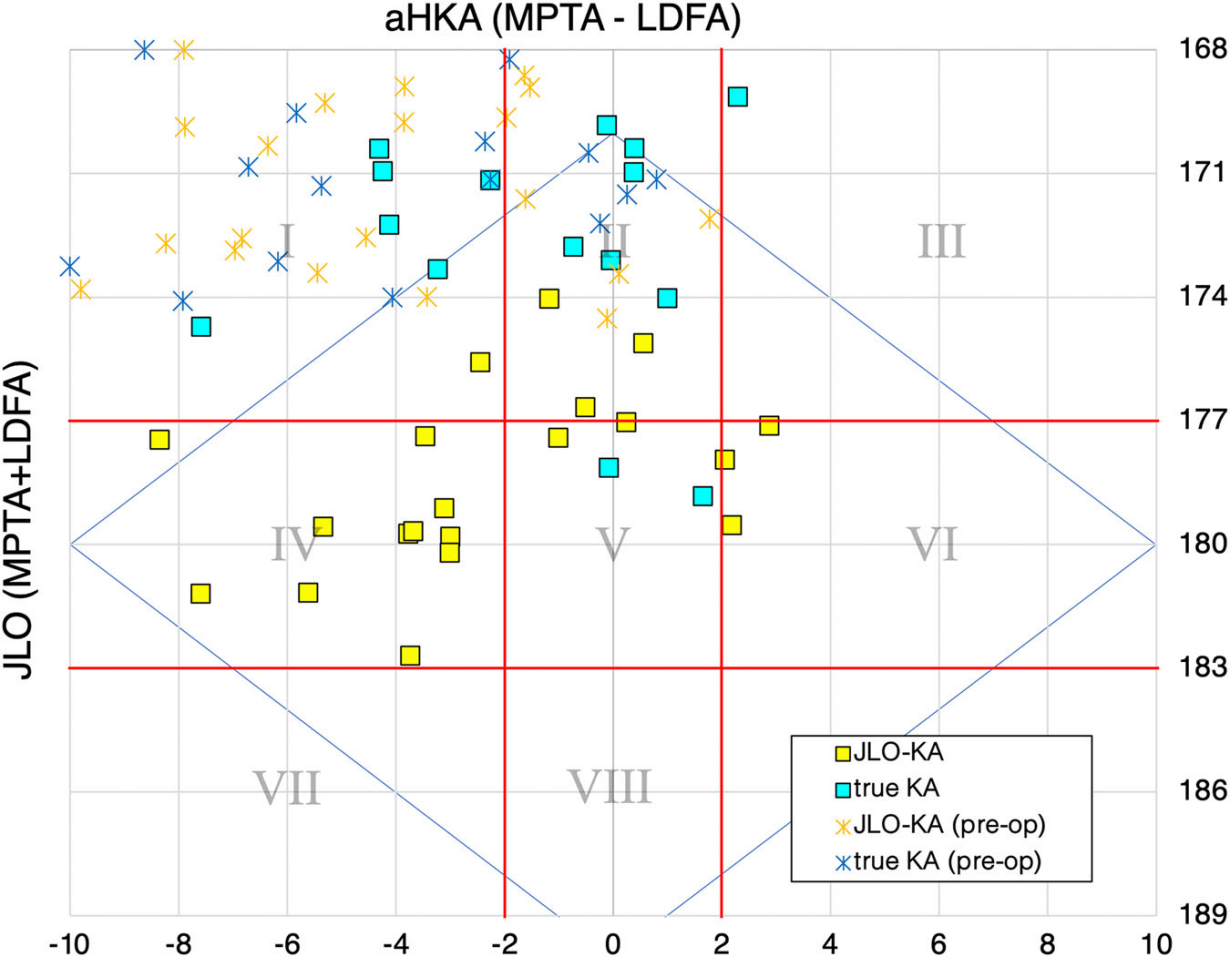
Distribution of aHKA and JLO on the CPAK grid after true KA and JLO‐KA. Postoperative scatter of aHKA (*x*‐axis) versus JLO (*y*‐axis). Compared with true KA (cyan), JLO‐KA (yellow) shifts cases from the apex‐distal band (CPAK I–III) toward neutral‐JLO (IV–VI) while aHKA distributions remain similar; the blue diamond indicates the rKA boundary (LDFA/MPTA 85°–95°) [3]. CPAK thresholds follow MacDessi et al. [19]. aHKA, arithmetic hip–knee–ankle angle; CPAK, Coronal Plane Alignment of the Knee; JLO, joint line obliquity; KA, kinematic alignment; LDFA, lateral distal femoral angle; MPTA, medial proximal tibial angle; rKA, restricted kinematic alignment.

On the CPAK grid (Figure 4), neutral‐JLO (Types IV–VI) occurred in 16/20 JLO‐KA knees (80.0%, 95% CI: 58.4–92.4) versus 2/15 true KA knees (13.3%, 95% CI: 3.7–37.9); difference +66.7 percentage points (95% CI: 20.5–88.2), *p* = 0.00013 (two‐sided Fisher's exact).

With respect to the first rKA boundary (LDFA and MPTA both within 85°–95°), 19/20 JLO‐KA knees (95.0%, 95% CI: 76.4–99.1) were within the boundary versus 7/15 true KA knees (46.7%, 95% CI: 24.8–69.9); difference +48.3 percentage points (95% CI: 6.5–74.3), *p* = 0.0019 (Fisher's exact).

### Clinical results

No patellar dislocations or component revisions occurred in either group within the first postoperative year.

## DISCUSSION

The most important finding of this study was that, in knees with pronounced apex‐distal JLO, JLO‐modified KA (JLO‐KA) produced a consistent shift of approximately 3° in component positioning—less femoral valgus, greater femoral external rotation, and less tibial varus—while preserving aHKA, all within the same calliper‐verified workflow used for true KA. Given that the alignment effect of reallocating the 2‐mm cartilage‐wear compensation had not been defined, this study quantified its magnitude and characterized the postoperative CPAK distribution. These intra‐articular changes are consistent with a more lateral position of the prosthetic trochlear groove [12], which is favourable for patellofemoral tracking [17, 26], and with limited medial tibial resection, which preserves medial tibial bone support [11, 23, 28, 31]. Collectively, these findings provide quantitative benchmarks for the planning, execution, and reporting of personalized alignment in apex‐distal configurations.

An apex‐distal JLO presents challenges for both MA and true KA in TKA [5, 12, 22, 23, 27]. One response is rKA, as proposed by Vendittoli et al., which constrains LDFA and MPTA each to 85°–95° (neutral ±5°) and, when necessary, adjusts aHKA toward neutral within ±3°. Long‐term outcomes with rKA are excellent at ≥10‐year follow‐up [3, 20]. Although procedurally distinct from rKA and applied without preset HKA targets or collateral releases, JLO‐KA generally positioned LDFA and MPTA within 85°–95° and shifted CPAK types from the apex‐distal row toward the neutral‐JLO row while avoiding apex‐proximal shifts (Figure 4). These findings suggest that reallocating cartilage‐wear compensation to the tibial side can reduce femoral valgus and tibial varus sufficiently to meet widely cited coronal alignment ranges [3].

JLO‐KA does not impose preset aHKA targets; instead, it uses selective intra‐articular adjustments to optimize component positioning while preserving soft‐tissue balance. Conceptually, this approach aligns with true KA as originally desctribed by Howell et al., for which excellent survivorship and clinical outcomes have been reported over 10–16 years of follow‐up without HKA constraints [8, 9]. Importantly, JLO‐KA does not aim to neutralize JLO (i.e., bring JLO to 180°); rather, it lateralizes the prosthetic trochlear groove and preserves medial tibial support while avoiding preset limb‐alignment targets. Whether this strategy is broadly applicable in populations in which apex‐distal alignment patterns are prevalent [15, 25, 32] warrants prospective evaluation with patient‐reported outcome measures (PROMs) and radiographic migration endpoints [31].

The clinical impact of changing CPAK type after TKA remains debated. Large series and a randomized trial have found no detriment in PROMs when the CPAK type changed under personalized alignment strategies [1, 2, 4]. By contrast, Konishi et al. identified two features associated with poorer outcomes: substantial shifts in HKA and postoperative apex‐proximal JLO; in their series, transitions from apex‐distal toward neutral JLO (I → IV or II → V) with preserved HKA were not associated with worse outcomes [14]. The pattern observed with JLO‐KA—reduced apex‐distal obliquity with maintained aHKA and clustering in the neutral‐JLO row (Types IV–VI)—is consistent with the latter scenario and supports a patient‐specific alignment approach [6].

Because JLO‐KA reallocates cartilage‐wear compensation to the medial tibial plateau, resulting in approximately 2‐mm elevation of the medial joint line, potential increases in mid‐flexion laxity warrant consideration. A simple geometric estimate suggests that, at 45° of flexion, the medial femoral condyle travels ≈2.8 mm ( = 2 × √2 mm) along its articular path, while the tibial surface is elevated by 2 mm, leaving a residual increase of ≈0.8 mm in the medial joint gap. Consistent with this, cadaveric testing showed that a 2‐mm joint line elevation increased mid‐flexion laxity, with larger increases at 4 mm [18].

In the true‐KA cohort, postoperative MPTA (85.6°) exceeded the preoperative value (83.5°) by ≈2°. A plausible explanation is restoration of tibial anatomy that was underestimated preoperatively because medial bone loss obscured the native joint surface [29]. As the extent of medial bone loss was not quantified in this series, this interpretation remains provisional.

## LIMITATIONS

This study has several limitations. First, the sample was small (*n* = 35) and derived from a single surgeon's retrospective series without an a priori power calculation; therefore, the findings should be interpreted as exploratory and hypothesis‐generating. Effect sizes with 95% CIs are reported to inform future studies. Second, technique allocation was not randomized but followed an implant‐dependent, stepwise adoption protocol, raising the possibility of selection and time‐trend (learning‐curve) confounding despite consecutive‐case capture. Third, implant designs differed between groups (ATTUNE/GMK Sphere for JLO‐KA; JOURNEY II/GMK Sphere for true KA), which may confound patellofemoral kinematics and clinical outcomes and preclude device‐matched comparisons. Fourth, 10 of 51 consecutive cases lacked postoperative CT and were excluded, introducing potential selection/attrition bias. Fifth, postoperative alignment was measured by a single experienced observer without formal intra‐ or interobserver reliability testing in this cohort; however, the same image‐matching workflow has been validated in prior studies [30, 33]. Sixth, follow‐up was limited to 1 year; longer term data on component migration, radiographic loosening, and patient‐reported outcomes are needed. Seventh, while the CPAK classification facilitates visualization of JLO and aHKA, it can obscure bone‐specific contributions (e.g., isolated femoral valgus vs. tibial varus); the functional knee phenotype framework [6] may provide complementary mechanistic insights.

### Future directions

Prospective comparative studies with standardized implants, dynamic assessments of patellar tracking, intraoperative soft‐tissue balance evaluation, and PROMs—ideally coupled with migration analysis—are warranted to determine whether the alignment changes achieved with JLO‐KA translate into improved anterior knee comfort, patellar stability, and tibial component longevity [12, 17, 22, 26, 31].

## CONCLUSION

Compared with true KA, JLO‐KA in apex‐distal knees produced approximately 3° reductions in femoral valgus and tibial varus and increased FCR while maintaining the aHKA, achieved by omitting femoral cartilage‐wear compensation within a calliper‐verified workflow.

## AUTHOR CONTRIBUTIONS

Tsutomu Maeda conceived the study and performed surgeries. Tsutomu Maeda and Mitsuhiko Kubo conducted the analysis. Theodore Derek Vernon Cooke provided conceptual guidance and manuscript revision. Shinji Imai and Kazutaka So contributed to interpretation and critical review.

## CONFLICT OF INTEREST STATEMENT

The authors declare no conflicts of interest.

## ETHICS STATEMENT

This study was approved by the Ethics Committee of Shiga General Hospital (No. 240521‐02), with opt‐out consent via public disclosure on the hospital website.

## Data Availability

The data sets generated and/or analysed during the current study are available from the corresponding author on reasonable request.
